# Scalable Enrichment of Immunomodulatory Human Acute Myeloid Leukemia Cell Line-Derived Extracellular Vesicles

**DOI:** 10.3390/cells10123321

**Published:** 2021-11-26

**Authors:** Heide-Marie Binder, Nicole Maeding, Martin Wolf, André Cronemberger Andrade, Balazs Vari, Linda Krisch, Fausto Gueths Gomes, Constantin Blöchl, Katharina Muigg, Rodolphe Poupardin, Anna M. Raninger, Thomas Heuser, Astrid Obermayer, Patricia Ebner-Peking, Lisa Pleyer, Richard Greil, Christian G. Huber, Katharina Schallmoser, Dirk Strunk

**Affiliations:** 1 Spinal Cord Injury and Tissue Regeneration Center Salzburg (SCI-TReCS), Cell Therapy Institute, Paracelsus Medical University (PMU), 5020 Salzburg, Austria; HMBinder@gmx.at (H.-M.B.); nicole.maeding@pmu.ac.at (N.M.); martin.wolf@pmu.ac.at (M.W.); andre.cronemberger@pmu.ac.at (A.C.A.); vari.balazs@oncol.hu (B.V.); linda.krisch@pmu.ac.at (L.K.); fausto.gueths@pmu.ac.at (F.G.G.); katharina.muigg@pmu.ac.at (K.M.); rodolphe.poupardin@pmu.ac.at (R.P.); anna.raninger@pmu.ac.at (A.M.R.); patricia.ebner@pmu.ac.at (P.E.-P.); 2 Department of Transfusion Medicine and SCI-TReCS, Paracelsus Medical University (PMU), 5020 Salzburg, Austria; k.schallmoser@salk.at; 3 Department of Biosciences, Paris Lodron University, 5020 Salzburg, Austria; constantin.bloechl@plus.ac.at (C.B.); Astrid.Obermayer@plus.ac.at (A.O.); c.huber@plus.ac.at (C.G.H.); 4 Vienna BioCenter Core Facilities GmbH, 1030 Vienna, Austria; thomas.heuser@vbcf.ac.at; 5 3rd Medical Department with Hematology, Medical Oncology, Rheumatology and Infectiology, Paracelsus Medical University, 5020 Salzburg, Austria; l.pleyer@salk.at (L.P.); r.greil@salk.at (R.G.); 6 Salzburg Cancer Research Institute (SCRI) Center for Clinical Cancer and Immunology Trials (CCCIT) and Cancer Cluster Salzburg (CCS), 5020 Salzburg, Austria; 7 Austrian Group for Medical Tumor Therapy (AGMT) Study Group, 1180 Vienna, Austria

**Keywords:** acute myeloid leukemia (AML), extracellular vesicles (EV), immunomodulation, MISEV

## Abstract

Acute myeloid leukemia (AML) cells can secrete trophic factors, including extracellular vesicles (EVs), instructing the stromal leukemic niche. Here, we introduce a scalable workflow for purification of immunomodulatory AML-EVs to compare their phenotype and function to the parental AML cells and their secreted soluble factors. AML cell lines HL-60, KG-1, OCI-AML3, and MOLM-14 released EVs with a peak diameter of approximately 80 nm in serum-free particle-reduced medium. We enriched EVs >100x using tangential flow filtration (TFF) and separated AML-derived soluble factors and cells in parallel. EVs were characterized by electron microscopy, immunoblotting, and flow cytometry, confirming the double-membrane morphology, purity and identity. AML-EVs showed significant enrichment of immune response and leukemia-related pathways in tandem mass-tag proteomics and a significant dose-dependent inhibition of T cell proliferation, which was not observed with AML cells or their soluble factors. Furthermore, AML-EVs dose-dependently reduced NK cell lysis of third-party K-562 leukemia targets. This emphasizes the peculiar role of AML-EVs in leukemia immune escape and indicates novel EV-based targets for therapeutic interventions.

## 1. Introduction

Acute myeloid leukemia is a genetically heterogeneous disease that originates after sequential acquisition of mutations and genomic aberrations resulting in clonal expansion of leukemia-initiating cells [1]. Common mutations target signaling and kinase pathway members, epigenetic modifiers, transcription factors, and tumor suppressors, including nucleophosmin 1 (NPM1), fms-like tyrosine kinase 3 (FLT3), rat sarcoma virus (RAS) protein family members, tumor protein p53 (TP53), and DNA methyltransferase 3 alpha (DNMT3A), representing prognostic markers involved in therapy stratification [2,3,4]. Although new forms of therapy, including small molecules, adoptive immune cell therapy and mutation-targeting agents are being applied, AML remains a disease with a high mortality due to a high rate of relapse, particularly in the elderly [5,6].

Extracellular vesicles (EVs) serve as evolutionary conserved regulators of cell and tissue biology and are considered to influence constitutive cellular process during development, homeostasis and regeneration, including hematopoiesis [7]. Different types of EVs, predominantly 30 to 1000 nm in size, are released by all cells. Historically, cell communication was considered to occur either cell-to-cell contact-dependent or contact-independent, via soluble factors, over distance. Secretion of EVs has been recognized as an additional major mechanism for cell-to-cell and cell-environment communication [8]. Early studies indicated already a key role of EVs in orchestrating immune responses [9,10]. The contribution of EVs to tumor progression and anti-tumor immunity is meanwhile well accepted [11,12]. Hypoxia, as an intrinsic property of certain tumor environments, can promote increased tumor-derived EV secretion [13,14]. Hypoxia can also induce elevated secretion of proangiogenic EVs by pluripotent stem cells indicating a more general mechanism [15]. In a community approach, minimal information for studies of EVs (MISEV) guidelines were established in 2014 and updated in 2018, recommending stringent reporting of EV isolation and characterization parameters, and encouraging the use of the ‘umbrella term’ EV for the various species of exomeres, exosomes, microvesicles, ectosomes, oncosomes, and apoptotic bodies [16,17].

In AML and myelodysplasia, an altered stromal stem cell niche can drive leukemia, and, vice versa, leukemia blasts can instruct a tumor-permissive environment that favors disease progression [18,19,20,21]. EVs were shown to contribute to the induction of a leukemia-permissive microenvironment by actively instructing stromal cells in the bone marrow [22]. It was also shown that EVs derived from primary AML bone marrow stromal cells transfer miR26a-5p to promote AML cell proliferation, migration, and marrow infiltration [23]. AML-EVs can transfer miR-150 and miR-155, targeting the cellular myeloblastosis (cMyb) protein, a key transcription factor to suppress hematopoietic stem/progenitor cell clonogenicity [24]. In addition, AML-EVs enforced quiescence in hematopoietic stem cells, thus suppressing healthy hematopoiesis in the leukemic niche [25]. Transforming growth factor beta (TGF-ß), enriched in EV preparations from AML patient sera, and EVs of the KASUMI-1 cell line suppressed NK-cell mediated cytotoxicity and down-regulated expression of NKG2D [26,27]. Other studies reported a contribution of AML-EVs to chemo-resistance [28,29]. The impact of AML-EVs on immune surveillance is, therefore, of strong interest. Particularly, the EV quantity, but also quality and characterization, remain paramount for preclinical and clinical research [8]. We took advantage of our protocols for stem cell and stromal cell EV isolation [15,30] and devised a scalable strategy for AML-EV purification.

## 2. Materials and Methods

### 2.1. Cell Culture and EV Isolation

AML cell stocks were purchased from American Type Culture Collection (ATCC; HL-60 ATCC-CCL-240 and KG-1 ATCC-CRL-8031) and the German Collection of Microorganisms and Cell Cultures (DSMZ; OCI-AML3 ACC582 and MOLM-14 ACC777). Cell lines were expanded in RPMI-1640 (Sigma-Aldrich, St. Louis, MO, USA) supplemented with 10% pooled human platelet lysate (HPL) [31], 5 mM N(2)-L-Alanyl-L-Glutamin (Dipeptiven, Fresenius Kabi, Bad Homburg, Germany), 100 U penicillin and 0.1 mg/mL streptomycin (PenStrep, Sigma-Aldrich) and 10 mM HEPES (Sigma-Aldrich) before cryopreservation in a working cell bank at −170 °C. For EV production and in indicated experiments, cells were expanded in RPMI-1640 containing 10% FBS (Gibco, Thermo Fisher Scientific, Waltham, MA, USA), 5 mM Dipeptiven, and 10 mM HEPES. K-562 cells were grown in RPMI-1640 supplemented with 10% FBS, 5 mM Dipeptiven, and 10 mM HEPES. After initial expansion, cells were transferred to RPMI-1640 supplemented with ITS+1 liquid media supplement (Sigma-Aldrich) as a serum replacement, 5 mM Dipeptiven, and 10 mM HEPES for two passages before initiating EV harvest in this medium.

Cells were expanded to cultures of 250 × 10^6^ cells/500 mL serum-supplemented medium before change to serum-free conditions. After three population doublings in RPMI/ITS+1, conditioned medium was harvested sequentially every 48–72 h for a maximum of six population doublings. Cells were separated from the conditioned medium by centrifugation at 300× *g* for 5 min at room temperature and reseeded at 0.5 × 10^6^ cells/mL. Cell samples for apoptosis assays and other flow cytometric analysis were stained immediately. Additional cell pellets, washed in PBS, were shock frozen and stored at −80 °C for later analysis (e.g., Western blot). Debris was removed from conditioned medium by centrifugation at 3000× *g* for 15 min. Conditioned medium was stored at −80 °C until further use.

Thawed and pooled conditioned medium was filtered plus concentrated using a 1600 cm^2^, 300 kDa cut-off, hollow fiber modified polyethersulfone (mPES) membrane filter column operated on a KR2i TFF System (Repligen, Waltham, MA, USA). The flow-through containing the virtually EV-depleted soluble factors (solF) was collected, aliquoted, and stored at −80 °C. The concentrated EV-containing medium was washed with 40-fold volume of sodium chloride 0.9% buffered with 10 mM HEPES to further deplete proteins (TFF1). TFF1 was further concentrated on a 20 cm^2^ column with a 300 kDa cut-off (TFF2). For proteomics, aliquots of TFF1 were further purified using 70 nm size exclusion chromatography columns (pEVsingle, Izon Science, Christ Church, New Zealand) [30]. Fractions 7, 8, and 9 were pooled as EV rich fraction (SEC EVs, high particle count measured by TRPS), and fractions 15–22 were pooled as protein-rich fraction (SEC control). Aliquots of all purification steps were stored at −80 °C for further analysis. Protein amount was measured using a detergent compatible (DC) protein assay (Bio-Rad, Hercules, CA, USA).

### 2.2. Particle Quantification

Particle concentration and size distribution of media, conditioned media, and different EV preparations was determined by TRPS. Samples were diluted in Dulbecco’s PBS containing 0.05% Tween 20 to an appropriate concentration for measurement on a qNano Gold (Izon Science) equipped with a NP150 nanopore (analysis size range 70–420 nm). For every sample, a minimum of 500 events was recorded. Measurements of particle-free medium (particle rate < 10/min) were stopped after 1 min. The instrument was typically operated with a stretch of 47 mm and an applied pressure of 10 mbar. Data were analyzed using Izon Control Suite software v3.4.

### 2.3. Transmission Electron Microscopy (TEM), Cryo-TEM, and Western Blot

TEM, cryo-TEM, proteomics, and Western blot were done as described [15,30].

### 2.4. Super-Resolution Microscopy

EVs (10 µL) were incubated for 48 h at 4 °C on Ibidi slides with 40 µL sodium chloride/HEPES. Staining was performed as previously described [15], using anti-human CD63-AlexaFluor488 (clone MEM-259, Invitrogen, Waltham, MA, USA, 0.2 mg/mL) and anti-human CD81-AlexaFluor647 (clone 454720, R&D Systems, Minneapolis, MN, USA, 0.2 mg/mL) for staining.

### 2.5. Immunomodulation Assay

Immune modulation assays were performed as previously described [32,33]. Briefly, PBMCs isolated from buffy coats of ten independent donors were pooled, labeled with carboxyfluorescein succinimidyl ester (CFSE) (Sigma-Aldrich) and stored in liquid nitrogen until further use. Stimulation of PBMCs was performed with either 5 µg/mL PHA (Sigma-Aldrich). EVs were added in ratios of 15,000:1, 5000:1, and 1666:1, EVs:PBMCs, respectively, based on previous titration [15,32,33]. The precise number of EVs secreted in vivo is not known. The highest EV:PBMC ratio was, therefore, considered to represent maximum secretion under stress conditions. Soluble factors were added in same volume as EVs. Cells were added in ratios of 1:1, 1:3 and 1:9 to the PBMCs, respectively. Antibody staining was performed using CD3-eF450 (eBioscienc™, Thermo Fisher Scientific, Waltham, MA, USA) to identify proliferating CD3^+^ T cells with excluding dead cells by fixable viability dye (FVD700; BD, Franklin Lake, NJ, USA) and cell debris. Acquisition was performed on a Gallios (Beckman Coulter, Brea, CA, USA) or LSR Fortessa (Becton Dickinson, Franklin Lakes, NJ, USA) flow cytometer. Data analysis was performed using Kaluza 2.1 software (Beckman Coulter) or FlowJo 10.7.1 (BD).

### 2.6. Cytotoxicity Assay

Cytotoxicity assays were performed using calcein release from pre-labeled K-562 target cells upon lysis [34]. We used unsorted PBMCs as effector cells resembling the composition of leukocytes found in peripheral blood more closely compared to sorted cells. In brief, K-562 cells were labeled with 10 mM calcein-AM (Thermo Fisher Scientific, Waltham, MA, USA) according to manufacturer’s instructions. PBMCs were incubated with AML-EVs for 24 h at EV:PBMC ratios 15,000:1, 5000:1 and 1666:1. As described above, these ratios were selected based on previous titration experiments. Afterwards, EV treated effector cells were co-cultured with labeled K-562 cells for 4 h at an effector-to-target ratio of 80:1. After 4 h, culture supernatants were collected and fluorescence was measured in a 96-well black plate (Thermo Fisher Scientific) on a plate reader at 520 nm (Tecan Spark, Tecan, Männedorf, Switzerland). Background lysis and maximum lysis were determined as release of calcein from cultured K-562 cells in the absence of PBMCs with (maximum lysis) or without (background lysis) 2% Triton X-100 (Sigma-Aldrich), respectively. Percent lysis was calculated as *%* lysis = (release − spontaneous release)/(maximum release − spontaneous release) × 100.

### 2.7. Flow Cytometry

AML cells were stained according to standard methods with antibodies against CD34-PECy7 (BD Pharmingen™, BD Biosciences, San Jose, CA, USA), HLA-DR-eF450 (eBioscienc™), CD13-FITC (Beckman Coulter), CD14-PE (BD), CD10-AF700 (BD), CD19-APC (BD), CD3-PECy7 (eBioscience™), CD4-AF700 (BD), CD8-PO (Invitrogen™), CD11c-PE (BD), CD45RA-PE-eF610 (eBioscience™), CD56-PE (eBioscience™), CD123-APC (eBioscience™), CD33-PE (BD), CD184-PE (BD), CD133-APC (Miltenyi Biotec, Bergisch-Gladbach, Germany), CD9-AF647 (R&D), CD63-AF647 (R&D), CD81-PE (molecular probes), CD49e-PE (BD), CD44-PE (BD), CD29-APC (BD), CD47-FITC (BD), CD172a/b- BV421 (BD), CD279-BUV395 (BD), CD73-eF450 (eBioscience™), CD39-BV421 (BD), CD86-PECy7 (eBioscience™), CD95-PE (BD), CD154-PE (BD), and live/dead stains 7-AAD (eBioscience™) and FVD520 (eBioscience™). After blocking in 5% sheep serum, cells were stained in 1% sheep serum for 20 min at 4 °C. Washed cells were acquired on a Gallios (Beckman Coulter) or a LSR Fortessa (Becton Dickinson) and analyzed using the Kaluza 2.1 (Beckman Coulter) or FlowJo v10.6 (Becton Dickinson) software.

For analyzing the apoptotic fraction of cultured cells, stainings were performed according to the manufacturer’s protocol using the annexin V apoptosis detection kit eF450 (eBioscience™) and 7-AAD or propidium iodide as live/dead stain. Cells were washed in cold PBS, rewashed in binding buffer, and stained for 15 min at room temperature for annexin V. Washed cells were resuspended in live/dead stain and analyzed immediately on the flow cytometer. Acquisition and analysis was performed alike antibody stainings.

AML-EV surface markers were analyzed using the MACSPlex Exosome Kit (Miltenyi Biotec) according to the manufacturer’s protocol: 5 × 10^8^ particles were incubated over-night with exosome capture beads before being washed and detected with anti-CD9, -CD63, and -CD81 antibodies conjugated to APC. Acquisition was performed using BD LSR Fortessa, and analysis was performed using FlowJo v10.6 (Becton Dickinson) and Excel (Microsoft).

### 2.8. Isolation of Extracellular Vesicles from Human Plasma Specimens

Plasma was obtained from venous blood of three high risk AML patients and three healthy donors. AML patient samples were provided from the biobank affiliated with the Austrian Registry of Hypomethylating Agents (clinicaltrials.gov identifier NCT01595295) [35,36,37]. Patients were 75 and 76 years old at blood biobanking and were selected for intermediate-to-poor cytogenic risk, as well as periperal blood blast counts >65%. Blood samples were centrifuged at 450× *g* for 10 min to obtain plasma. After collection into fresh sample tubes, plasma was centrifuged for 15 min at 3000× *g* to remove cellular debris. Samples were aliquoted and stored at −80 °C until further processing.

For EV isolation, plasma samples were thawed, centrifuged at 2500× *g* for 15 min, and transferred to fresh tubes. EVs were isolated using SEC. To this end, 70 nm SEC columns (qEV10, Izon Science) were equilibrated with 120 mL PBS supplemented with 0.9% sodium chloride and 10 mM HEPES (SEC buffer). Subsequently, 5 mL plasma were loaded onto the column, and—after release of the void volume (20 mL)—eighteen 3 mL fractions were collected, covering the peak EV and protein fractions. Collected fractions were analyzed for particle content using TRPS. The three fractions with the highest particle content (fraction range #2–#5) were pooled and concentrated to 1.2–2.17 × 10^11^/mL for further use in functional assays. Concentration was carried out by loading samples onto 100 K MWCO Spin-X^®^ UF concentrators (Sigma-Aldich) and subsequent centrifugation at 2000× *g* for 2–15 min (depending on sample concentration). Isolated and concentrated plasma EVs were used in cytotoxicity assays as indicated.

### 2.9. TMT Proteomics and Bioinformatic Analysis

Preliminary TMT proteomics data of KG-1 samples was performed as stated in Reference [30]. Briefly, peptide samples were prepared employing S-trap columns (Protifi, Huntington, NY, USA) according to the manufacturer’s instructions. Digested peptides (12.5 µg) of cell pellet, conditioned medium (CM), soluble factors (solF), SEC EVs, and SEC control were labeled using a TMT 10-plex™ kit (Thermo Fisher Scientific). Separation was carried out on a nano-HPLC instrument (UltiMate™ U3000 RSLCnano, Thermo Fisher Scientific). Separation of TMT-labeled samples was performed on a 2000 mm µPAC™ C18 column (PharmaFluidics, Ghent, Belgium). All mass spectrometry measurements were conducted in positive ion mode on a hybrid mass spectrometer (QExactive™ Plus benchtop quadrupole-Orbitrap^®^ mass spectrometer) equipped with a Nanospray Flex™ ion source (both Thermo Fisher Scientific) and a SilicaTip™ emitter with 360 µm outer diameter, 20 µm inner diameter, and a 10 µm inner tip diameter (New Objective, Woburn, MA, USA). Multiplexed proteomics sample (1.0 µL, 2 µg of peptides) was injected once using the microliter pick-up mode (loop volume 5.0 µL). All data were evaluated using MaxQuant software (version 1.6.12.0) using default settings. A protein list was obtained from the Uniprot database including Swiss-Prot entries for homo sapiens (access: 30 March 2020) and was provided for MaxQuant searches [38,39]. TMT-labeled data were further processed using Perseus software package (version 1.6.12.0) [40]. Only protein groups with all channels quantified were included for analysis, log2-transformed and normalized by subtraction of the median. Further analysis of the TMT-labeled samples was conducted using Ingenuity^®^ Pathway Analysis (IPA, Qiagen Bioinformatics, Hilden, Germany).

### 2.10. Statistics

All statistical tests were performed using GraphPad Prism version 7.03 (GraphPad Software, San Diego, CA, USA). Statistically significance was tested using Student’s t-test or analysis of variance (ANOVA), depending on applicability. Differences were considered significant when *p* was <0.05.

## 3. Results

### 3.1. AML-EV Isolation

**Figure 1:**
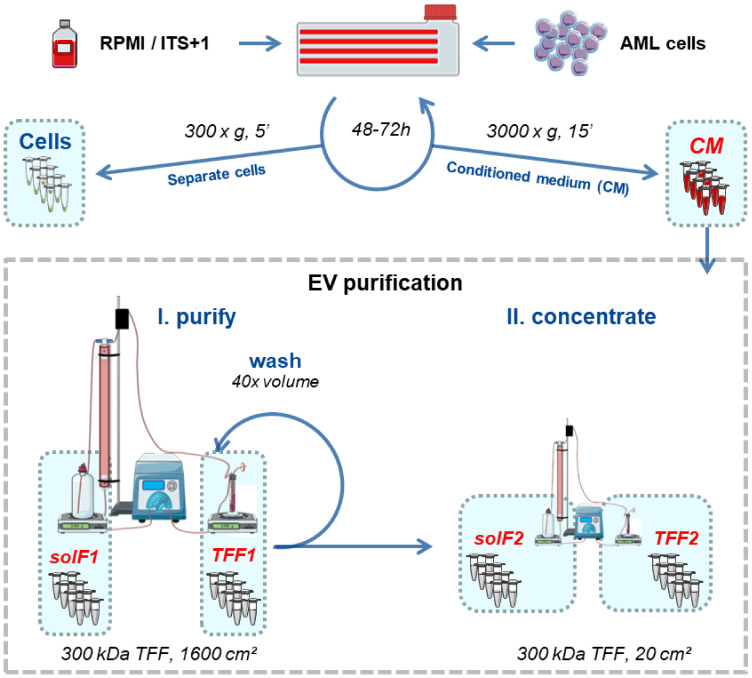
Schematic workflow for serum-free large-scale EV production from AML cell lines. Cells were cultured for 48–72 h intervals in RPMI/ITS+1. AML cells (viability ≥ 85%) were separated by centrifugation (300× *g*, 5 min, RT), and the conditioned medium (CM) was further centrifuged (3000× *g*, 15 min, RT) to remove cell debris and large vesicles in advance of EV isolation. We counted cells and reseeded 0.5 × 10^6^ cells/mL for up to five consecutive rounds, to harvest at least 2 L of CM. The pooled CM was subjected to tangential flow filtration (TFF; 300 kDa column) to separate EVs (TFF1) from soluble factors (solF1). The remaining protein and lipid contaminants were removed from TFF1 EVs by washing with 40 × the concentrated volume of buffer in the same TFF1 run. The remaining solution was concentrated on a smaller 300 kDa column to harvest purified EVs (TFF2). Graphic created with Servier Medical Art [41].

**Figure A1:**
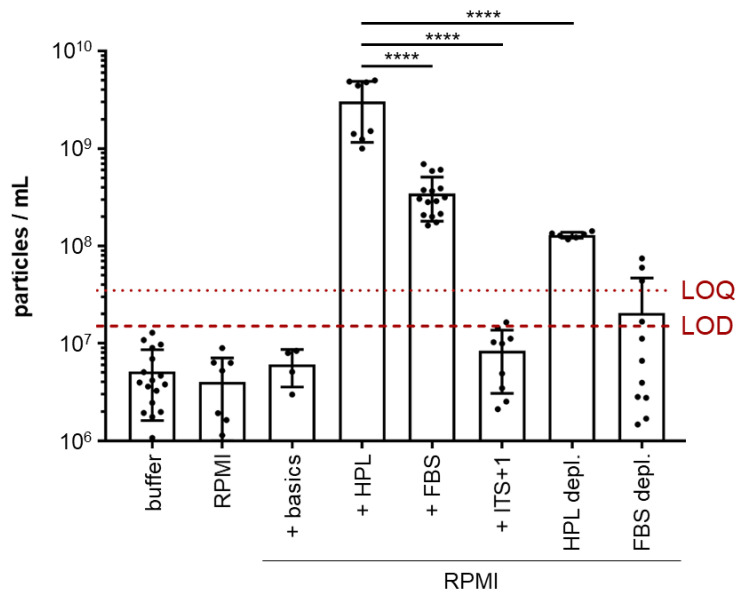
Particle content varies among common culture media for cell-derived EV analysis. Particle counting was performed using tunable resistive pulse sensing (TRPS) of common media compositions of (i) unsupplemented RPMI, (ii) RPMI supplemented with Dipeptiven and HEPES (+ basics) and serum supplemented media (iii) without depleting serum particles (10% human platelet lysate (+ HPL) or 10% fetal bovine serum, (+ FBS) or iv.) with depleting serum particles with a 500 kDa column size by tangential flow filtration (TFF; +HPL depl., +FBS depl.). The dotted red line marks the limit of quantification (LOQ, calculated as mean of replicate measurements of buffer (PBS + 0.025% Tween 20) + 9 × SD of mean, mean 3.58 × 10^7^ particles/mL). The dashed red line marks the limit of detection (LOD, calculated as mean of replicate measurements of buffer (PBS + 0.025% Tween 20), +3 × SD of mean, mean 1.53 × 10^7^ particles/mL). Serum-free media (ITS+1 supplement replacing serum) showed particle counts below LOQ in unconditioned medium, making it feasible to study EVs produced by AML cells. One-way ANOVA, **** *p* < 0.0001; *n* = 6–14.

**Figure A2:**
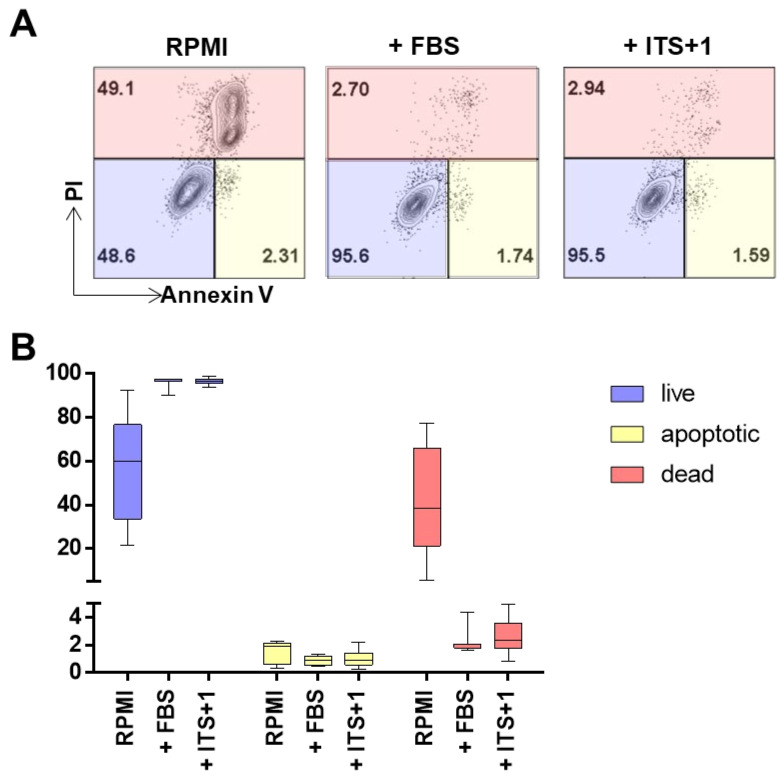
Monitoring cell death in defined culture conditions for AML cells. Defined media conditions were used to culture AML cell lines for at least five passages before harvesting and assessing apoptosis by flow cytometry. Culture conditions varied accordingly: *RPMI* defines RPMI complemented with Dipeptiven, HEPES and antibiotics; *FBS* defines basic medium supplemented with 10% heat inactivated FBS and ITS+1 defines basic medium supplemented with 1X ITS+1. (**A**) Dot plots depict the percentage of gated events for live, dead and apoptotic cells for the different culture conditions. A representative example of cell line KG-1 is shown. Events gated as live show negative staining for propidium iodide (PI, *y*-axis) and annexin V-eF450 (*x*-axis); dead events refer to the percentage of the population that is single positive for PI and apoptotic refers to the percentage of the population of annexin V single positive events. (**B**) Quantification of the flow cytometry analysis depicts live populations as blue bars, dead events in red and the percentage of apoptotic cells in yellow bars. (Details are described in the Section 2; *n* = 4 per culture condition, 3 technical replicates; 10,000 events/replicate).

We selected four AML cell lines representing key mutations, as well as peripheral blood and bone marrow origin, and immature versus mature phenotype, respectively (Table A1 in Appendix A). Cell line identity, purity and viability was assessed by multicolor flow cytometry (Figure S1). For subsequent characterization of AML-EVs, we validated the particle content in standard media, confirming previous experience that serum-supplemented media contain high amounts of particles which could mask cell-derived EVs during further analysis [30]. To avoid interference of serum- or platelet-derived EVs in supplemented media with downstream analysis, we selected a serum replacement which showed only minor particle contamination (Figure A1). AML cell lines cultured with this serum replacement (insulin-transferrin-selenium+linoleic acid, ITS+1) showed viability and apoptosis rates comparable to conventional fetal bovine serum (FBS)-supplemented cultures. All four cell lines could be propagated for culture periods of 10–14 days in ITS+ supplemented media. Significant apoptosis was observed in cultures without FBS and without ITS+1, arguing against the common EV harvest strategy in media just devoid of serum. ITS+1 supplementation allowed expansion of AML cell lines over extended periods with just minute apoptosis rates comparable to FBS-supplemented media (Figure A2). Based on these preliminary data, we set up a standardized workflow for large-scale manufacturing of AML-EVs (Figure 1).

### 3.2. AML-EV Quantification and Characterization

**Figure 2:**
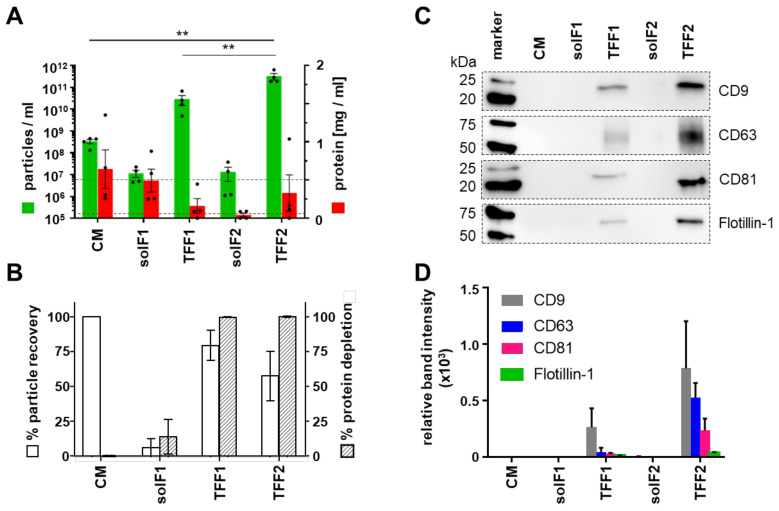
AML-EV purification process using sequential TFF. AML cell lines were cultured in a particle-poor RPMI medium using ITS+1 as serum replacement. (**A**) Particle concentration was measured with tunable resistive pulse sensing (TRPS) using a 150 nm pore (green bars). Protein amount was measured using a detergent compatible (DC) protein assay (red bars). Limit of detection (LOD) of the TRPS measurement using the 150 nm pore is depicted as a green dotted line. The red dotted line represents the LOD of the DC protein assay. Fractions analyzed as indicated (conditioned medium, CM; soluble factors, solF, cycles 1 and 2; tangential flow filtration EV-containing retentate, TFF, cycles 1 and 2). (**B**) Recovery compared to total amount of input particles showing mean 89.97% recovery after TFF1 purification and mean 66.23% recovery of total particles after TFF2. The recovery of particles within the solF fractions was less than 5% for solF1 and solF2 suggesting a loss of EVs in the column during TFF2. (**C**) Representative Western blot analysis of identical sample volumes (20 µL) of EV purification fractions of MOLM-14 EVs. (**D**) Quantification by densitometry showing significant enrichment of EV-markers CD9, CD63, CD81 and flotillin-1 by TFF. (**A**,**B**), *n* = 4, one-way ANOVA, ** *p* < 0.01; (**C**,**D**), *n* = 2).

To monitor purification efficiency of leukemia cell line-derived EVs, protein concentration and particle amount was assessed using a detergent compatible (DC) protein assay and tunable resistive pulse sensing (TRPS). Conditioned media contained 3.02 ± 0.64 × 10^8^ particles/mL (mean ± SEM). The two cycles of TFF termed TFF1 and TFF2, resulted in a significant consecutive EV enrichment, to 2.52 ± 1.08 × 10^10^ and 3.21 ± 1.05 × 10^11^ particles/mL, respectively, accompanied by an efficient protein depletion in TFF1 (Figure 2A). The recovery rate was calculated as percentage of total particles obtained after TFF enrichment (TFF2) [15,30] compared to total particles contained in the conditioned medium (Figure 2B). EV identity was confirmed by immunoblotting of proteins enriched in EVs, including tetraspanins CD9, CD63, and CD81, and the caveolae-associated integral membrane protein flotillin-1 (Figure 2C). Densitometry of western blots normalized to total protein loaded (stain-free gel) showed up to 26.28-fold enrichment in small EV-enriched CD81 in TFF1 and up to 235.75-fold in TFF2 preparations, compared to the CD81 band intensity derived from loading conditioned medium, respectively (Figure 2D).

**Figure 3:**
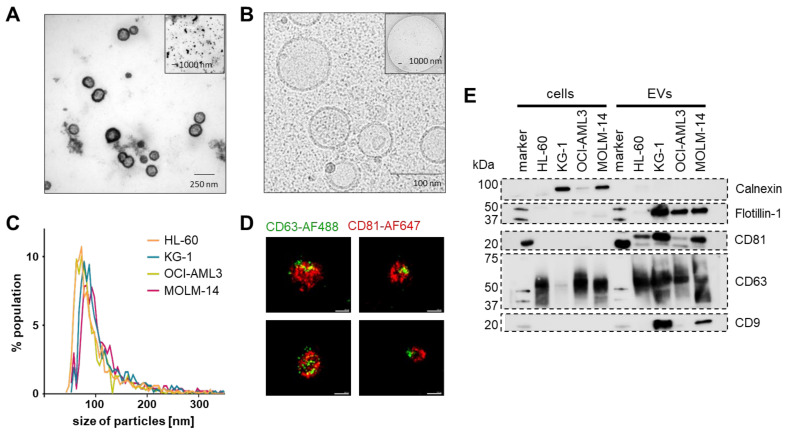
Purified EV identity. (**A**) Representative negative contrast electron microscopy of MOLM-14 EVs. (**B**) Representative cryo-electron microscopy showing EVs with typical double-membrane structures. (**C**) Mode size distribution using a 150 nm pore on a tunable resistive pulse sensing (TRPS) device (*n* = 3 measurements per cell line). (**D**) Representative super-resolution microscopy images (dSTORM mode, ONI) of MOLM-14 EVs stained with an anti-human CD63-AlexaFluor488 and an anti-human CD81-AlexFluor647 antibody. Scale bars represent 100 nm. (**E**) Western blots of identity markers according to the MISEV 2018 criteria suggesting absence of cellular contaminants in the EV purifications by lack of calnexin (90 kDa) and enrichment in small EV markers CD9 (25 kDa), CD63 (40–60 kDa), CD81 (26 kDa) and flotillin-1 (48 kDa). *n* = 3, representative blots shown.

Ultrastructure of the isolated EVs was confirmed by negative contrast TEM (Figure 3A) and double-membrane morphology by cryo-TEM (Figure 3B). Size measurement by TRPS using a 150 nm nanopore showed a mean overall size of EVs purified from the four cell lines of 79.36 ± 11.07 nm (HL-60-EVs = 82.67 ± 12.02, KG-1-EVs = 82 ± 4, OCI-AML3-EVs = 74 ± 2.88, MOLM-14-EVs = 79.67 ± 4.91; mean ± SEM; *n* = 3 per cell line) (Figure 3C). Representative super-resolution microscopy of MOLM-14-EVs depicted CD63 and CD81 co-localization (Figure 3D). In order to monitor EV-associated proteins according to MISEV2018 criteria [16], western blotting was performed for the four cell lines in parallel to the corresponding purified EVs. The absence of the endoplasmic reticulum protein calnexin confirmed EV purity. Calnexin was detected in the cell fractions except for HL-60, where it was previously found among the lowest expressed genes (−0.85) in a gene set analysis [42]. Small EV identity proteins flotillin-1 and the tetraspanins CD63 and CD81 were enriched in the EV fractions showing a typical heterogenous size pattern. Flotillin-1 showed only a minute signal in HL-60 EVs. CD9 was detected at high levels only in KG-1 and MOLM-14 derived EVs, despite higher surface expression level on OCI-AML3 cells (Figure 3E and Figure S1).

**Figure A3:**
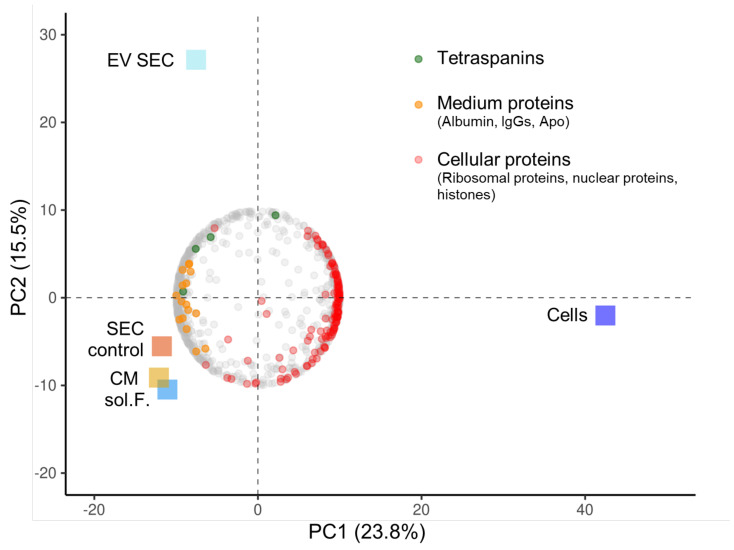
Tandem mass tag proteomics of AML EV fractions. Principle component (PC) analysis biplot showing clustering of the different samples (squares) and the loadings of the 906 variable proteins (circular dots; representing the weights for each original variable—here proteins—when calculating the PC). PC1 showed the strongest separation between cells and other samples (23.8% variance explained) while PC2 separated the size exclusion chromatography-enriched KG1-EVs (EV SEC) from other samples (15.5% variance explained). Protein-rich SEC fraction (SEC control), conditioned media (CM) and soluble factors (sol. F.) were the most similar samples. Individual loadings showed enrichment of tetraspanins (green circles) in the EV SEC fraction. KG1 cells were enriched for cellular proteins (red circles; including histones, ribosomal and nuclear proteins). The cluster “SEC control/CM/sol. F.” was enriched for presumably culture medium-derived proteins (orange circles; albumin, IgGs and apolipoproteins). Five KG-1-derived sample fractions as indicated by colored boxes; mean data, fractions analyzed in duplicate.

To further investigate the AML-EV surface marker signature, we used MACSPlex technology as described [43] and found a reproducible display of tetraspanins and adhesion molecules, such as the hyaluron receptor CD44 and the fibronectin receptors CD49e and CD29, respectively. Hematopoietic markers expressed on EVs reflect the identity regarding the parental leukemic cell lines, as described previously [43,44] (Figure S2). A comprehensive summary of the adherence to MISEV guidelines [16,17] within this study is shown in Table A2. TMT proteomics showed a strong separation of cellular proteins from SEC-EV-associated tetraspanins and less clear separation of medium proteins (Figure A3).

### 3.3. AML-EV Immunosuppressive Function

**Figure 4:**
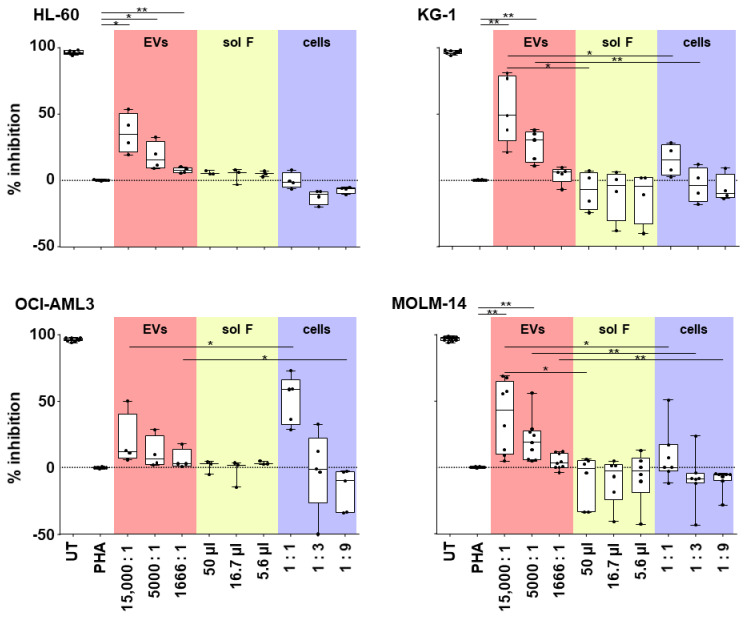
EVs derived from AML cells suppress T-cell proliferation. Purified AML derived EVs (red fields) were compared to the EV-producing cells (blue fields) and the EV-depleted soluble factors (sol F; yellow fields) for their capacity to inhibit T-cell mitogenesis in a dose-dependent manner. Abbreviations: UT, untreated peripheral blood mononuclear cells (PBMCs), from healthy donors; PHA, phytohemagglutinin-stimulated PBMCs. Paired Student’s *t*-test, * *p* < 0.05, ** *p* < 0.01; *n* = 3–8, dots represent individual experiments.

To study functional differences in the immune response mediated by AML cells, EVs, and soluble factors, an immune modulation assay was performed as previously described for stromal cells and their EVs [32,33]. T-cell proliferation was measured by flow cytometry after 4-day incubation of CFSE-labeled and PHA-stimulated PBMCs in the absence or presence of increasing doses of AML-EVs, soluble factors, and corresponding AML cells, respectively. EVs from all four cell lines showed a significant dose-dependent inhibition of CD3^+^ T cell proliferation compared to PHA-stimulated PBMCs without EV treatment. At the highest dose, KG-1-EVs inhibited T-cell proliferation at mean 53.07%, followed by MOLM-14-EVs (mean 38.52%), HL-60-EVs (mean 35.5%), and OCI-AML3 EVs (mean 20%). Interestingly, the AML cells and their secreted soluble factors showed significantly less if any inhibition, except for OCI-AML3 cells at highest dose (1:1, AML cells:responder PBMCs). A measurable but not significant enhancement of T cell proliferation was observed in response to soluble factors derived from KG-1 and MOLM-14, as well as lower doses of OCI-AML3 cells (Figure 4).

**Figure 5:**
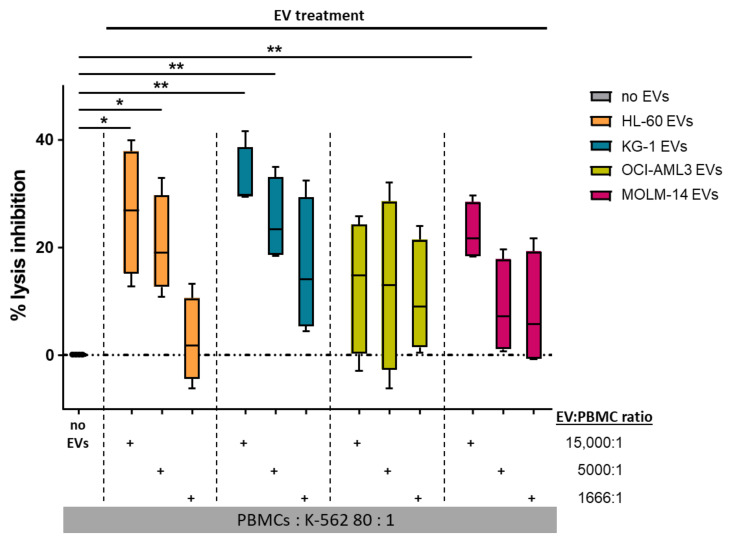
AML-EVs inhibit NK-mediated cytotoxicity. PBMCs were treated with different doses of AML-EVs before analysis of their capacity to lyse K-562 leukemia target cells. Treatment with graded doses of purified EVs from all four AML cell lines as color-coded in the legend. Abbreviations: EVs, extracellular vesicles; PBMCs, peripheral blood mononuclear cells from healthy donors. Paired Student’s *t*-test, * *p* < 0.05, ** *p* < 0.01; *n* = 4.

**Figure A4:**
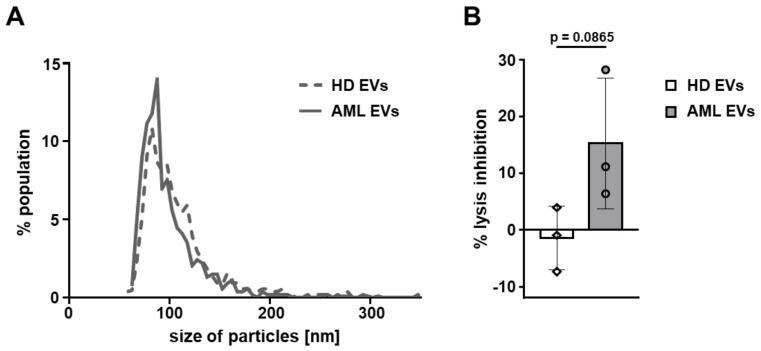
Size distribution and inhibitory capacity of EVs isolated from human plasma. EVs were isolated from plasma of AML patients and healthy donors (HD) using size exclusion chromatography (SEC). (**A**) Mode size distribution of primary EVs as determined by TRPS using a 150 nm pore. (**B**) Inhibition of K-562 target cell lysis by PBMCs treated with primary EVs at a ratio of 15,000:1 EVs:PBMCs. Abbreviations: AML, acute myeloid leukemia; EVs, extracellular vesicles; HD, healthy donor; PBMCs, peripheral blood mononuclear cells from healthy donors. (Unpaired Student’s *t*-test; *n* = 3 independent donors, symbols represent individual donors).

Since decreased cytolytic activity of natural killer (NK) cells is a prominent feature of AML [45], we sought to investigate the effect of AML-EVs on NK-mediated cytotoxicity. PBMCs were incubated with three different doses of AML-EVs (EV: PBMC ratio 15,000:1, 5000:1, and 1666:1) for 24 h followed by a 4 h co-incubation of PBMCs with calcein-loaded NK target cells K-562. NK-mediated lysis was determined as the release of calcein from K-562 corresponding to the level of cell death after co-incubation. EVs from all cell lines but OCI-AML3 significantly inhibited NK-mediated lysis over the untreated control (no EVs) in a dose-dependent manner. Treatment with the highest EV dose (15,000:1) resulted in a mean ± SEM inhibition of 26.63% ± 5.81 (HL-60), 32.64% ± 2.98 (KG-1), 13.13 ± 6.26 (OCI-AML3), and 22.83% ± 2.71 (MOLM-14). Notably, there appeared to be cell line-related differences in the potency of the EVs. While OCI-AML3 EVs displayed variable but low capacity to inhibit NK cytolytic capacity, KG-1 EVs were on average almost 3-fold as effective (Figure 5). In a limited number of samples, we also confirmed that AML-EVs derived from leukemic patient plasma inhibited K-562 lysis by third party NK cells (Figure A4).

## 4. Discussion

In this study, we devised a standardized high-content isolation strategy for AML-EVs using four established representative cell lines. The expanded AML cells and their soluble factors were separated, in addition, for comparison. Following MISEV2018 guidelines [16], the AML-EV quantity, quality and function were characterized. Using an optimized serum-free and particle-reduced culture medium enabled isolation of virtually pure AML-EVs. Starting from 2780–6600 mL conditioned medium allowed obtaining high quantities of AML-EVs of up to 1.31 × 10^13^ particles (e.g., from KG-1) per batch. EV morphology was confirmed by negative contrast TEM and cryo-TEM. The mode size of the isolated AML-EVs showed only limited variability between mean 74–83 nm, consistent with published data [46]. Efficient depletion of culture-derived protein was demonstrated, as well as the EV identity and enrichment, by Western blotting and super-resolution microscopy. The separation of EVs from the EV-producing cells and their EV-depleted secretome permitted direct functional comparison of these fractions. We found that AML-EVs but not the AML cell line-derived soluble factors and also not the cells themselves inhibited T cell proliferation. Thus, isolated AML-EVs from all four cell lines significantly inhibited the lysis of K-562 leukemia target cells in a dose-dependent manner. We also confirmed previous results [26] showing the capacity of primary AML patient plasma-derived EVs to inhibit K-562 lysis, compared to healthy donor plasma-derived EVs.

Immune evasion is one of the hallmarks of tumor progression [47]. Better understanding of the process of AML-EV-induced immunomodulation may enable therapeutic interventions to improve AML outcome. The Whiteside group was the first to demonstrate inhibition of NK cell-mediated lysis of K-562 cells by EVs isolated from patient serum in 2011 [26]. They further demonstrated that the AML patient serum-derived EVs targeted purified healthy donor-derived NK cells directly resulting in down-regulation of NKG2. TGF-ß1 was shown to mediate decreased SMAD phosphorylation and reduced function of NK cells in their model [26]. They later extended their findings by showing that AML patients receiving NK-92 therapy blocked the anti-leukemic cytotoxicity of the NK-92 cells, resulting in a lack of response. Mechanistic side studies indicated binding of AML-EVs to the therapeutic NK-92 cells but no signs for EV uptake. Instead, signaling via surface receptors was concluded to be responsible for the lack of therapeutic efficiency during the adoptive cell therapy [27]. The same group further showed that chemotherapy significantly increased the secretion of AML-EVs, thereby potentially contributing to therapy resistance [48].

Our data showing a dose-dependent inhibition of NK cell-mediated lysis of leukemic cell by AML-EVs derived from four independent cell lines confirmed these observations. Surprisingly, despite intensive literature search, we were not able to identify additional data sets demonstrating inhibition of NK cell lysis of AML cells by AML-EVs. Our preliminary results using a limited number of patient samples confirmed the observations by the Whiteside group. Additional research is, thus, required to challenge extended reproducibility of these data regarding the EV-mediated trophic immune escape mechanisms in AML and beyond. Circumventing EV-mediated immune escape, e.g., by blocking tumor EV release or inhibiting their uptake may represent a novel therapeutic concept. Heparin was shown to inhibit cellular uptake of EVs by binding to EVs, causing their aggregation and reducing their binding to recipient cells [49,50]. Heparan sulfate can act as receptor for human glioblastoma EVs. Dependent on its sulfation pattern, size and charge, heparin was found to act as a competitor for heparan sulfate binding of EVs, efficiently blocking their uptake [51]. Not just the conventional unfractionated heparin but also low molecular weight heparins were able to reduce the migration of human pancreatic carcinoma cells, induced by EV-containing malignant pleural effusion in an animal model [52]. Additional animal studies showed heparin-induced reduction of tumor cell adhesion and inhibition of tumor growth and metastasis by low molecular weight heparins, but the overall survival of tumor patients was not increased by heparin therapy [53,54]. More standardized study protocols and a dose-response relationship would be required to define hypothetic anti-neoplastic clinical effects of heparins particularly related to EV uptake [53]. The benefits of prophylactic or therapeutic heparin medication for thromboembolism or sepsis-induced disseminated intravascular coagulation, particularly related to corona virus disease (COVID19) are currently discussed and investigated in clinical trials [55].

The clinical impact of EVs covers a much wider spectrum of applications, including immunotherapy and drug delivery, in addition to their use as diagnostic and prognostic biomarkers [8]. Over the last decade, particularly translation of EV-based biomarkers from bench to bedside was initiated [56]. Sophisticated technology is now in place using miRNA-containing EVs as biomarker for breast cancer [57]. Significant progress enabled more precise estimation of physiologic and pathologic blood EV counts [58]. Heterogeneity of the manifold EV populations, particularly those derived from healthy and malignant hematopoietic cells, still is an issue [7], but a consensus exists that EVs represent the next generation of biomarkers [59]. EV biomarker studies are not restricted to blood or plasma but may address any bodily fluid, such as urine [60]. Standardization guidelines are already in place [61,62].

Recognizing the role of EVs as mediators of paracrine signaling during cell-based therapies, despite lack of engraftment of the transplanted cells, resulted in the postulation of novel therapeutic concepts based on the assumption that EVs can partly replace allogeneic cell therapies [63]. EVs have also been shown to function during virtually all stages of cancer progression [64]. The efficiency of EVs as a cell-free vaccine made of dendritic cell-derived EVs was already shown in sophisticated experimental models more than two decades ago [11]. A growing number of clinical trials involving EVs is currently ongoing [8]. Furthermore, high hopes relate to drug targeting via EVs, including RNA-based medicines, in the foreseeable future [65].

The scalable workflow for AML-EV isolation established in this study may, thus, serve as a technology supporting further research toward therapeutic targeting of the deleterious immune escape effects of AML-EVs. Our observation that AML-EVs but not the AML cells or their secreted soluble factors also inhibit T cell proliferation might relate to the higher level of complexity of the immunosuppressive functions of AML-EVs [66]. Both T cell- and NK cell-based functional assays may serve as screening readouts to identify druggable targets. Hydroxy-methyl-glutaryl-coenzyme A reductase (HMGCR), the rate-limiting enzyme of cholesterol synthesis, was recently identified as one candidate target involved in elevated AML-EV release during chemotherapy [48]. Inhibition of HMGCR by high dose pravastatin during idarubicin plus cytarabine therapy of relapsed/refractory AML in a phase II study did not meet criteria for a positive study based on the response rate (*p* = 0.062), but results were considered encouraging [67]. Screening additional drug candidates in well-standardized in vitro assays [68] using the AML-EV isolation technology described in our study may, thus, help to identify or re-purpose molecules which can be tested clinically for blocking the immune escape effects of AML-EVs.

## Data Availability

Not applicable.

## Supplementary Materials

The following are available online at https://www.mdpi.com/article/10.3390/cells10123321/s1, Figure S1: Flow cytometry phenotype of AML cell lines; Figure S2: AML EV surface marker characterization by bead-based flow cytometry.

## Appendix A

**Table A1:** Comparison of AML cell lines used in this study. Listed are the common name, typical mutations, AML type, French-American-British (FAB) classification [69]. Abbreviations: c-Myc, human cellular homolog of avian Myelocytomatosis virus gene; NRAS, neuroblastoma RAS viral oncogene homolog; NPM1, nucleophosmin 1; DNMT3A, DNA-Methyltransferase 3A; FLT-3, fms-like tyrosine kinase 3.

Cell Line	Mutations	Tumor Type	FAB Class	Source
**HL-60**	c-myc	acute promyelocytic leukemia	M2	peripheral blood
**KG-1**	NRAS	acute myelogenous leukemia	M1	bone marrow
**OCI-AML3**	NPM1, DNMT3A	acute monocytic leukemia	M4	peripheral blood
**MOLM-14**	FLT-3	relapse of MDS patient	M5a	peripheral blood

**Table A2:** Adherence to MISEV2018 criteria for research with EV material. Comments regarding EV nomenclature, isolation, characterization, collection, and storage conditions, as well as functional studies, were summarized.

Nomenclature				
***Use of generic term EV for AML-EVs***				
**Collection and storage**				
**Releasing cell information**		**cell type and origin**	Acute myeloid leukemia (AML) EVs from four cells lines (HL-60, MOLM-14, OCI-AML3 and KG-1)	
		passaging	6 doublings	
		seeding density	*0.5 × 10^6^ cells/mL*	
		culture volume	*2780–5933 mL (HL-60); 4220–6220 mL (KG-1); 3170–6600 mL (OCI-AML3); 5276–5695 mL (MOLM14)*	
		culture vessel	*2528 cm^2^ (CF4)*	
Culture conditions		culturing medium	*RPMI/10% FBS; 2 final passages RPMI/ITS+1*	
		time of cultivation	*48–72 h × 5 for pooling conditioned medium*	
		harvesting medium	*RPMI supplement with ITS+1 (serum free)*	
		time of cultivation	*10–14 days*	
		cell count at harvest	*2.5 × 10^6^ cells/500 mL*	
Storage and recovery		conditioned medium	*Storage temperature: −80 °C*	
		EV preparations	*storage temperature: −80 °C/snap freeze*	
**Isolation**				
Differential centrifugation		*300× g for 5 min, SX4750, Allegra X-12R, Beckman Coulter* *3000× g for 15 min, SX4750, Allegra X-12R, Beckman Coulter*		
Tangential flow filtration, TFF		*TFF1: followed by washing with 40 volumes of buffer (sodium chloride + 10 mM HEPES), column surface area: 1600 cm^2^, pore size: 300 kDa; TFF2: TFF1 concentration, 20 cm^2^, 300 kDa column*		
**Characterization**				
		Parameter	Unit	Method
Quantification	Size & concentration	particle number (TFF2)	1.87–3.89 × 10^11^ particles/mL (HL-60); 6.29–22 × 10^11^ particles/mL (KG1); 2.79–9.52 × 10^11^ particles/mL (OCI-AML3); 1.88–3.87 × 10^11^ particles/mL (MOLM14)	*TRPS*
		particle size (mode)	82.67 ± 12.02 nm (HL-60); 82 ± 4 nm (KG1); 74 ± 2.88 nm (OCI-AML3); 79.67 ± 4.91 nm (MOLM14)	*TRPS*
	Composition	protein content	435–721 µg/mL (HL-60); 1037–2300 µg/mL (KG-1); 118–1444 µg/mL (OCI-AML3); 99.5–287 µg/mL (MOLM14)	*DC protein assay*
Identity (proteins)		trans membrane	*CD9, CD63 and CD81*	*WB*
		cytosolic, EV recovered	*Flotillin-1*	*WB*
		cell compartment	*Calnexin*	*WB*
Visualization		*Transmission electron microscopy*		
		*Super-resolution microscopy (CD63-AF488/CD81-AF647)*		
**Functional studies**				
Immunomodulation assay		Dose-dependent effect of EVs on CD3 T cell proliferation		
Cytotoxicity assay		Does-dependent effect of EVs on NK-mediated K-562 lysis		
