# Supplementary material for: Scalable Enrichment of Immunomodulatory Human Acute Myeloid Leukemia Cell Line-Derived Extracellular Vesicles
Source: Cells. 2021 Nov 26;10(12):3321. doi: 10.3390/cells10123321 (PMC8699161; doi:10.3390/cells10123321)
Supplement: Supplementary file 1 [file cells-10-03321-s001.zip › cells-1449943-supplementary.pdf]

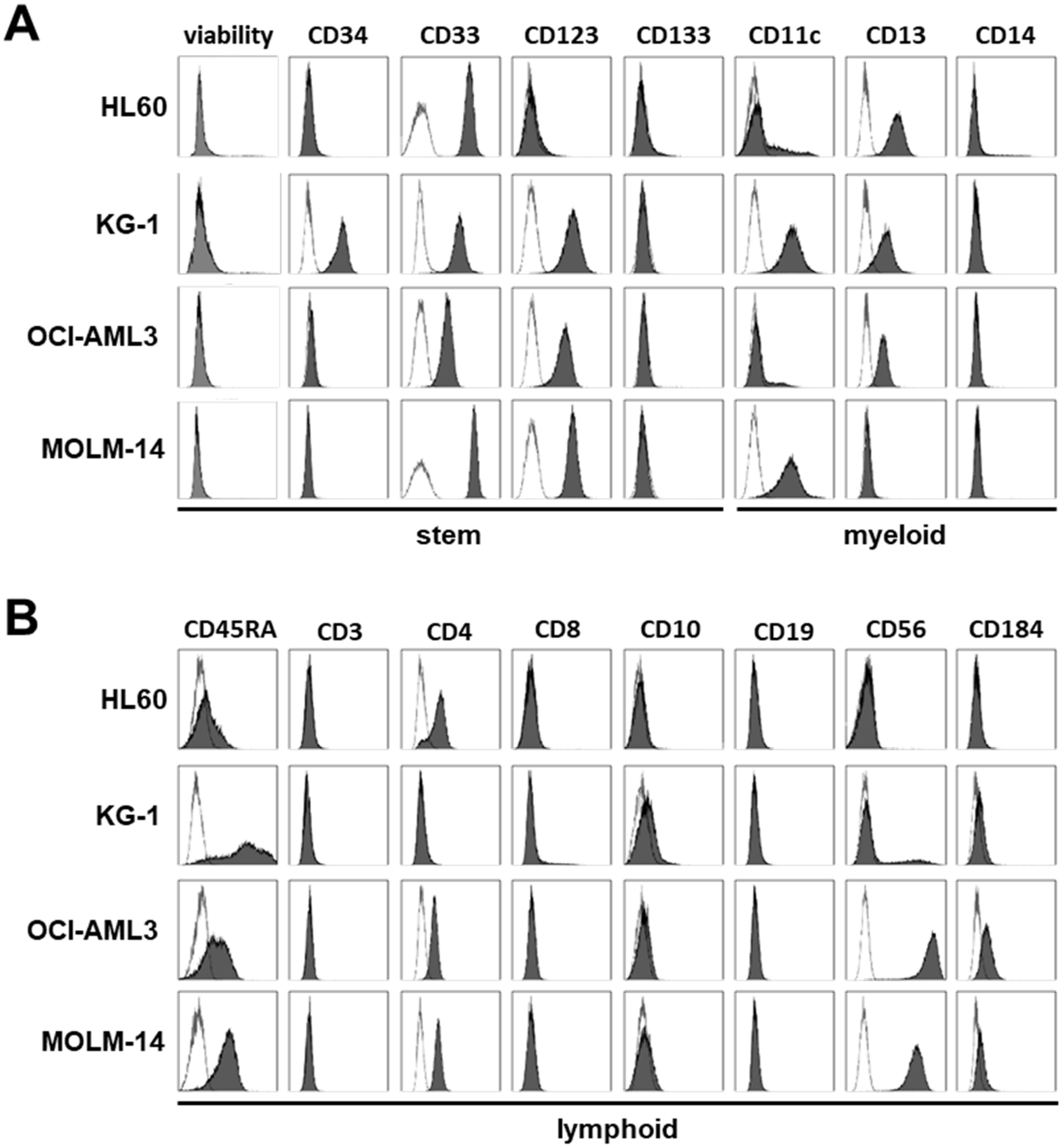
A
viability
CD34
CD33
CD123
CD133
CD11c
CD13
CD14
HL60
KG-1
OCI-AML3
MOLM-14
stem
myeloid
B
CD45RA
CD3
CD4
CD8
CD10
CD19
CD56
CD184
HL60
KG-1
OCI-AML3
MOLM-14
lymphoid

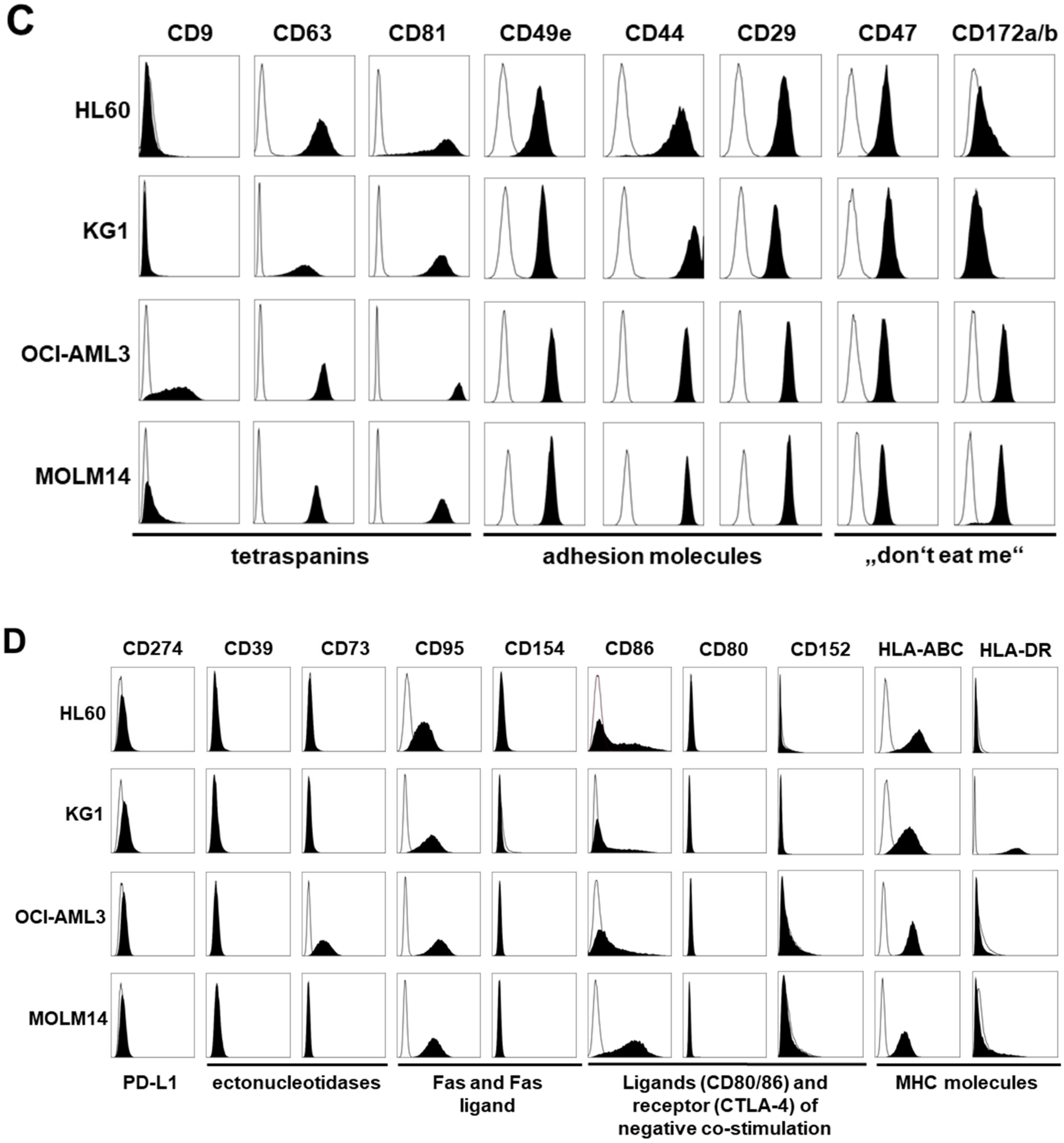

Figure S1. Flow cytometry phenotype of AML cell lines. To confirm the immune phenotype, flow cytometric analysis of AML cell lines stained with selected directly fluorescently labeled antibodies (gray filled histograms) and the corresponding isotype controls (open histograms) was performed. (**A**) Stem cell and myeloid markers. (**B**) Lymphoid markers. (**C**) Tetraspanins, adhesion molecules, "don't eat me" signal CD47 and signal regulatory proteins CD172a/b. (**D**) Immune regulatory molecules PD-L1 (CD274), 5′ ectonucleotidases (CD39, CD73), Fas/ligand, co-stimulatory molecules and MHC class I and II.

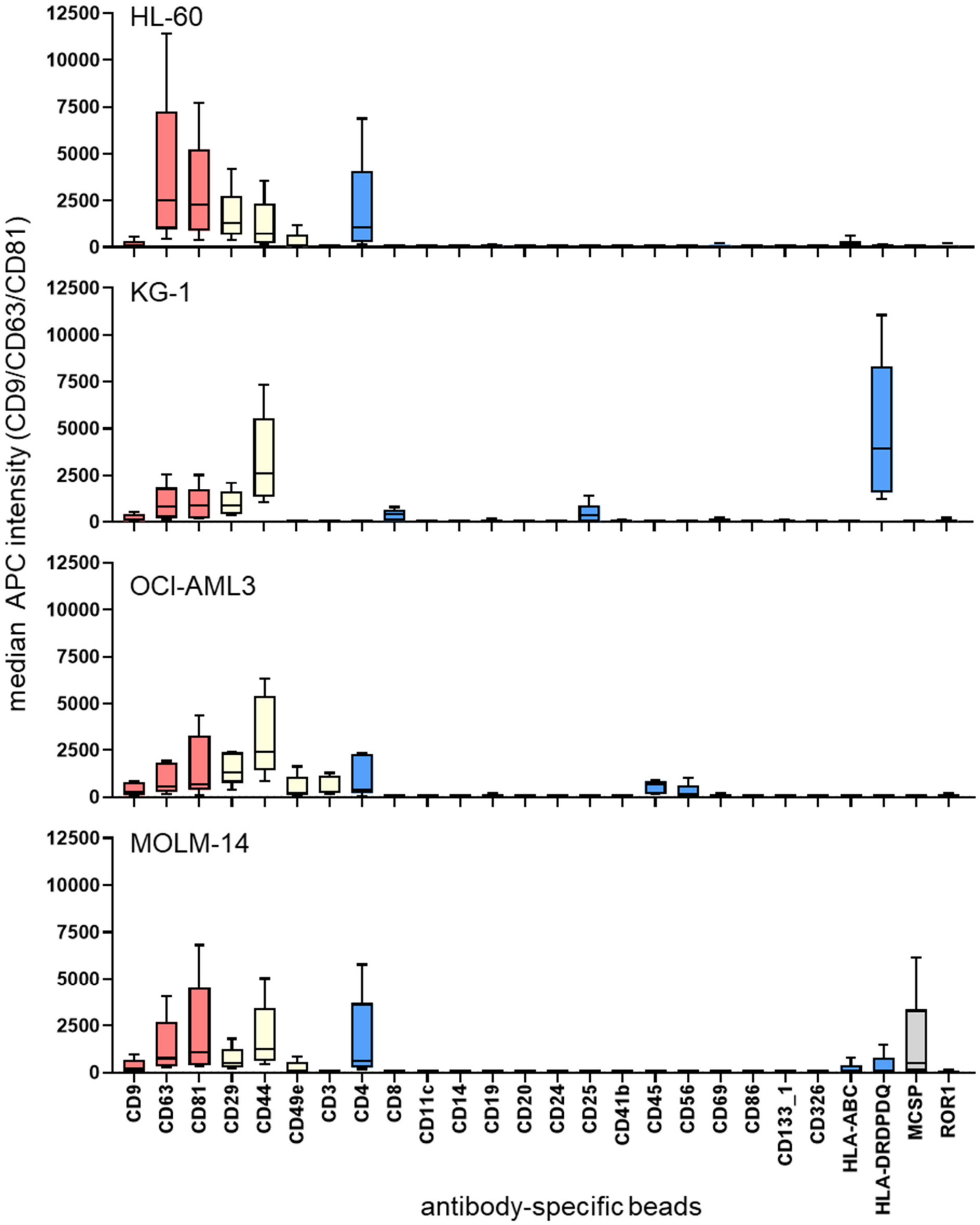

Figure S2. AML EV surface marker characterization by bead-based flow cytometry. Incubating purified EVs with antibody-coated polystyrene beads (x-axis) and staining with an APC-conjugated pan-tetraspanin antibody (normalized median APC intensity depicted on the y-axis) shows the presence of tetraspanins (red bars) CD63 and CD81 and to a lesser extent CD9 on the surface of EVs from all AML cell lines. In addition, adhesion proteins (yellow bars) CD29, CD44 and CD49e were detected on the surface of purified EVs. Hematopoietic markers (blue bars) reflect the surface markers on cells of different AML subtypes (n = 3).

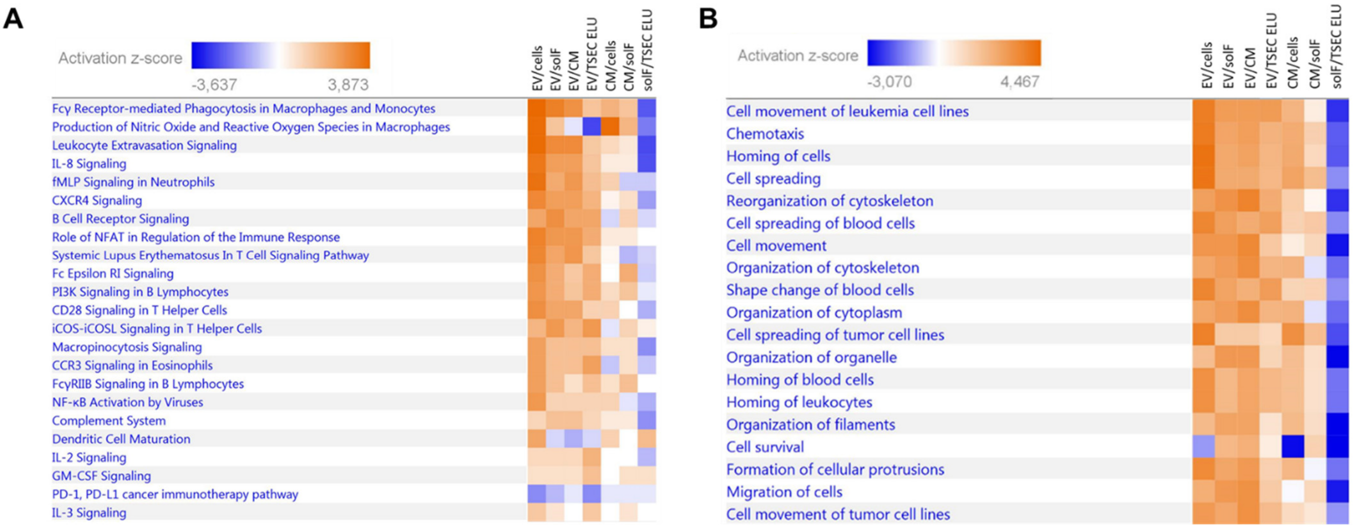

Figure S3. Quantitative tandem mass tag proteomics. A) Immune response and B) cancer-related pathways were enriched in proteins identified in EVs over proteins found in cells or the soluble protein fraction.
